# Clinical and Molecular Differentiation Between Malignant Rhabdoid Tumor of the Kidney and Normal Tissue: A Two-Case Report

**DOI:** 10.3389/fonc.2021.659709

**Published:** 2021-03-30

**Authors:** Chenghao Zhanghuang, Shuo Chen, Li Li, Zhen Yang, Yucheng Xie, Jiwei Li, Haoyu Tang, Xiaoli He, Liuyi Dong, Bing Yan

**Affiliations:** ^1^Department of Urology, Kunming Children’s Hospital, Kunming, China; ^2^Department of Pharmacology, Anhui Medical University, Hefei, China; ^3^Yunnan Key Laboratory of Children’s Major Disease Research Kunming Children’s Hospital, Kunming, China; ^4^Department of Oncology, Kunming Children’s Hospital, Kunming, China; ^5^Department of Pathology, Kunming Children’s Hospital, Kunming, China

**Keywords:** malignant rhabdoid tumor of kidney, RNA-sequencing, KEGG, GO, child preschool, BioSystems

## Abstract

**Background:**

Malignant rhabdoid tumor of the kidney (MRTK) is a rare type of tumor that lacks typical clinical manifestations. Herein, we presented clinical data of 2 children with MRTK. In addition, we used a high-throughput RNA-sequencing (RNA-seq), GO analysis, and KEGG signaling pathway analysis to examine gene expression differences at the transcripts level between 2 patients with MRTK and 3 patients with non-tumor diseases without other symptoms.

**Case report:**

Preoperative B-scan ultrasonography and computed tomography (CT) examination in 2 cases suggested nephroblastoma. Both patients were treated with radical nephrectomy. After the operation, MRTK was confirmed by pathological examination. Child 1 and Child 2 then received 7 courses and 12 courses of regular chemotherapy, respectively. Child 1 was followed up for 2 years, and Child 2 for 3.1 years without showing symptoms. RNA-seq results showed 2203 differential genes (DEGs) in the kidney tissue of children with MRTK compared to normal tissue (p <0.01). GO analysis suggested that most DEGs participate in protein binding. KEGG results showed that the DEGs were mainly involved in the PI3K-Akt signaling pathway and microRNA-related proteins.

**Conclusion:**

The PI3K-Akt signaling pathway and microRNA-related proteins as targets have extremely high potential value for the diagnosis and treatment of MRTK.

## Introduction

Malignant rhabdoid tumor of the kidney (MRTK), a rare type of malignant rhabdoid tumor (MRT), is a highly aggressive tumor that occurs in infants and young children. The tumor has a poor prognosis, and the incidence rate in men is slightly higher than in women (1.5:1) (1). The overall 3-year survival rate in patients with MRTK ranges from 12% to 38.4%; it is the highest in children aged 24 months or older and the lowest in those between 0 and 5 months of age (1, 2). In China, a recent study reported that within a 5-year follow-up, out of 35 cases, one survived (3). In addition, in children younger than 6 months, this condition is often accompanied by distant metastases, including metastases to the brain (4). Surgery plus postoperative radiotherapy and chemotherapy is the main approach to treat patients with MRTK. Yet, so far, there is still no uniform standard for the treatment of MRTK.

The disease was first reported in 1978 by Beckwith et al. (5) and Haas et al. (6). MRTK has the same characteristics as all MRTs. Patients with MRTK have an abnormal expression of the tumor suppressor gene SMARCB1/INI-1 (7–9). Moreover, the detection of SMARCB1 gene mutation has been associated with a worse prognosis (1). At the same time, cytogenetic studies have also found that most MRTK patients have chromosome 22 monomer deletion or 22q111.2 hSNF/INI1 gene mutation inactivation. This, in turn, leads to loss of INI-1 protein expression in the nucleus (10), which is of great significance as it can be differentiated from renal rhabdomyosarcoma.

MRTK lacks typical clinical manifestations. Most children develop abdominal masses, hematuria, or abdominal pain as the first symptoms. The tumor is often misdiagnosed as a Wilms tumor. B-scan ultrasonography can be used as a preliminary screening test, but it lacks specificity. Recent studies have found that CT and magnetic resonance imaging (MRI) can be useful for detecting early small tumors with subrenal hematoma/effusion (4, 11). Agrons et al. found that two-thirds of MRTK patients have dark crescent-shaped areas; the MRTK is unilateral and single, while Wilms Cell tumors can be bilateral or multiple (12). Preoperative imaging examination can be performed to distinguish Wilms tumor from renal blastocytoma. The final diagnosis needs to be confirmed by SMARCB1 gene detection or immunohistochemical INI-1 negative.

There are various signs, which indicate that the occurrence and development of MRTK are closely related to epigenetics (13). In this study, we used RNA-seq technology to investigate the transcription level of children with MRTK. The current report on MRTK mainly focused on the study of related proteins of traditional hSNF/INI1 and other genes, ignoring other information at the whole RNA level. The aim of this study was to screen new possible targets related to the occurrence and development of MRTK through global detection of RNA level, which can provide new ideas and theoretical basis for the prevention, diagnosis, and treatment of MRTK.

## Materials and Methods

### Intraoperative Pathological Sample Acquisition

Intraoperative pathological tissues from 2 MRTK patients and 3 patients with non-tumor diseases without other symptoms were collected and analyzed after their family signed the informed consent. After identification by the pathology department, the normal tissue area in the intraoperative pathological tissue of children with non-tumor diseases without other symptoms was taken as a control. The central area of the tumor taken by MRTK children was used as the pathological sample of MRTK. After collection, save the sample in the RNA storage solution.

### High-Throughput RNA-Sequencing (RNA-Seq) Analysis

The total RNA was extracted using TRIzol reagent. RNA-Seq was conducted according to a previously reported method (14). Briefly, after quality control and purification, the total RNA from each sample was used to enrich mRNA. The mRNA was fragmented and reverse-transcribed to cDNA. The cDNA was then purified, linked with the adaptor, size selected, and PCR amplified to obtain the cDNA library. After the quality check, libraries were sequenced through the Illumina platform.

Clean reads were obtained by using the preprocessing tool Trimmomatic (v0.36) to remove adapters and low-quality reads from the raw sequencing reads. Clean reads were mapped to Ensembl GRCh38 reference genome using the alignment program TOPHAT2. The mapped data were assembled into transcripts and quantified to obtain a matrix of expression values in FPKMusing StringTie v1.3.1c. Differentially expressed genes (DEGs) were screened out according to the logarithmic value of gene expression fold change (FC) between the PBS-treated cells and the OxLDL-treated cells. If logFC>1 or <1, and there was a significant statistical difference, it was judged as aDEGs. The distribution of the volcanic map of the DEGs was obtained by R software, and functional annotations were performed using Ensembl GRCh38 annotation files. Sequencing data have been uploaded from the GO database (https://www.ncbi.nlm.nih.gov/GO/query/acc.cgi?acc=GSE167547).

### Gene Ontology (GO) Construction and Kyoto Encyclopedia of Genes and Genomes (KEGG) Pathway Analysis

To explore the potential interactions between all genes at the protein level, online retrieval tool GO (https://GO.org/) was used. The KEGG pathway analysis was performed using online biotool KEGG Mapper 83.0 (http://www.kegg.jp/kegg/mapper.html).

### Statistical Analysis

The experimental data were analyzed using the R toolkit. A *P-*value <0.05 was considered to be statistically significant.

## Results

### 1. Case Description

#### Review of the Course of 2 Renal Malignant Rhabdoid Tumors

The study was approved by the Ethics Committee of Kunming Children’s Hospital.

##### Case 1

Child 1 was a 1.8-year-old girl who was admitted to the hospital due to painless naked hematuria that lasted 5 days. The family of the child reported the presence of dark brown urine that lasted for 5 days. The child had no frequent urination and no abdominal pain. On November 28, 2018, a urine routine examination at the first people’s Hospital of Xundian City indicated increased red blood cells in the urine, after which the patient was transferred to our hospital. B-scan ultrasonography suggested the left lower kidney solid mass sonogram (considering nephroblastoma). Since the onset of hematuria (November 29, 2018), the patient had a normal stool, a slight increase in urine, and no significant body weight change.

**Figure 1:**
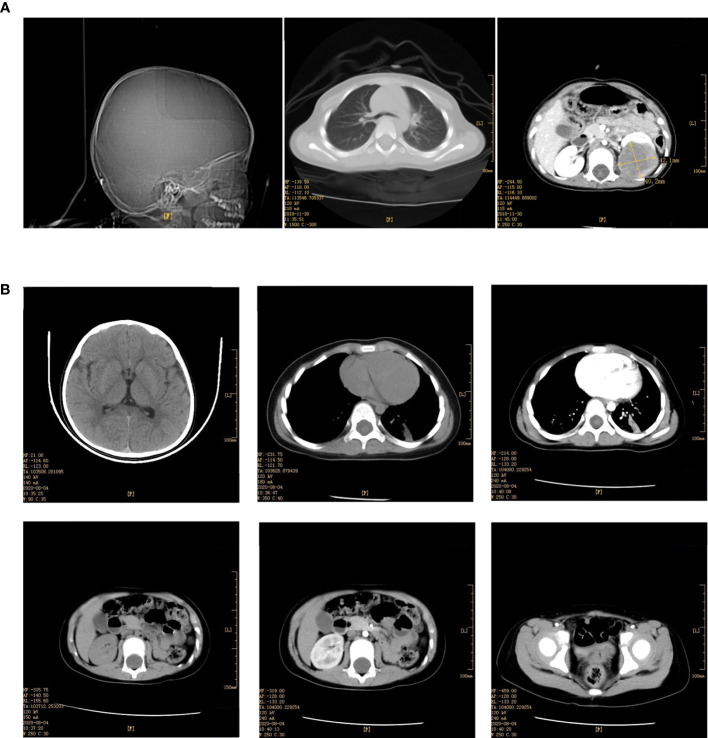
**(A)** Enhancement of skull, chest, whole abdomen CT plain scan before the operation (December 03, 2018). **(B)** Enhancement of skull, chest, whole abdomen CT plain scan after the operation (August 04, 2020).

Blood and fecal routine, biochemistry testing, coagulation function, and tumor markers were negative, including human chorionic gonadotropin (HCG), alpha-fetoprotein (AFP), and carcinoembryonic antigen (CEA). Urine red blood cells were 40355.9/UL, the cortisol (for measuring adrenal function) was 1262 nmol/L, and the adrenocorticotropic hormone was 305.6 pg/ml. Enhanced CT scan of the head, chest, and abdomen (**Figure 1A**) suggested a round-soft tissue density in the middle and lower part of the left kidney, CT about 40 HU, size of approx. 4.4 cm×3.9 cm×4.8 cm with a clear border. The enhancement scan showed mild to moderate uneven enhancement. There was no obvious abnormality in the head and lungs. The bilateral hip joint and sacrococcygeal X line were both normal.

Radical resection of the left renal tumor was performed under general anesthesia on December 7, 2018. The size of the renal region was about 7.5 cm×5.0 cm×4.5 cm cystic mass. The boundary between tumor and residual renal tissue was clear. There were adhesions between the tumor and the ipsilateral adrenal gland, peritoneum and inferior vena cava, and a varicose artery’s nutritional tumor tissues in the renal hilum. The operation was successful, the operation time was 130 min, and the intraoperative bleeding was 60 ml without blood transfusion.

**Figure 2:**
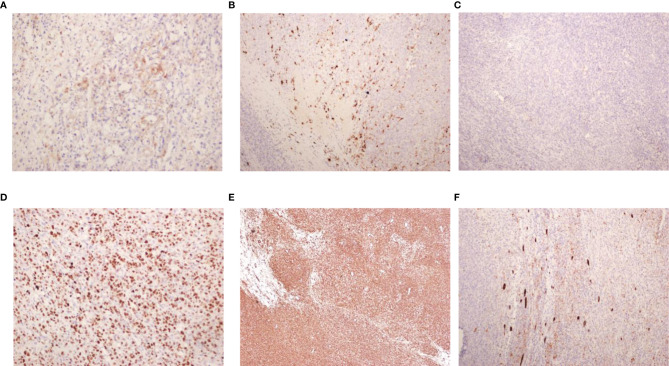
Pathological findings. **(A)** EMA (x200); **(B)** S-100 (x100); **(C)** INI-1 (x100); **(D)** Ki-67 (x200); **(E)** Vim (x40); **(F)** CK (x100).

Under histological examination (**Figure 2**), the tumor cells showed patchy diffuse distribution. The tumor cells were round in shape, short fusiform, with a big nucleus and bubbles. Eosinophilic vitreous inclusion bodies were found in some cytoplasm. Nuclear fission and multiple necroses were also observed. No tumor components were found in 4 lymph nodes of the ureteric stump, perirenal fat, perirenal, and retroperitoneal. Immunohistochemistry results were the following: INI-1(-), CyclinD1(+), CK(+), EMA(+), S-100(+), Ki-67 hot spot 80%(+), Vimentin(+), Desmin(-), CD10(-), Myogenin(-), MyoD1(-), TFE3(-). The pathological results were: (left renal tumor) combined with immunohistochemical results. Thus, MRTK was confirmed. No tumor components were found in ureteral stumps, perirenal fat, and lymph nodes. According to the National Wilms Tumor Study of the American Association for the Study of Nephroblastoma NWTS Standards, it was considered as clinical stage II. The child recovered and was discharged 7 days after the operation. She received further treatment in Shanghai Fudan Children’s Hospital: carboplatin 16.7”.mg/(kg·d) on Day1 and Day2, etoposide [vp163.3 mg/(kg·d) on Day1-3, and cyclophosphamide [CTX14.7 mg/(kg·d) on Day1-5” regimen chemotherapy once a day. She received 7 rounds of chemotherapy in total. The treatment was completed on June 27, 2019. The most recent follow-up (August 4, 2020) suggested no metastasis (**Figure 1B**). At present, 2 years and 1 month after surgery, the patient continues to be asymptomatic.

##### Case 2

**Figure 3:**
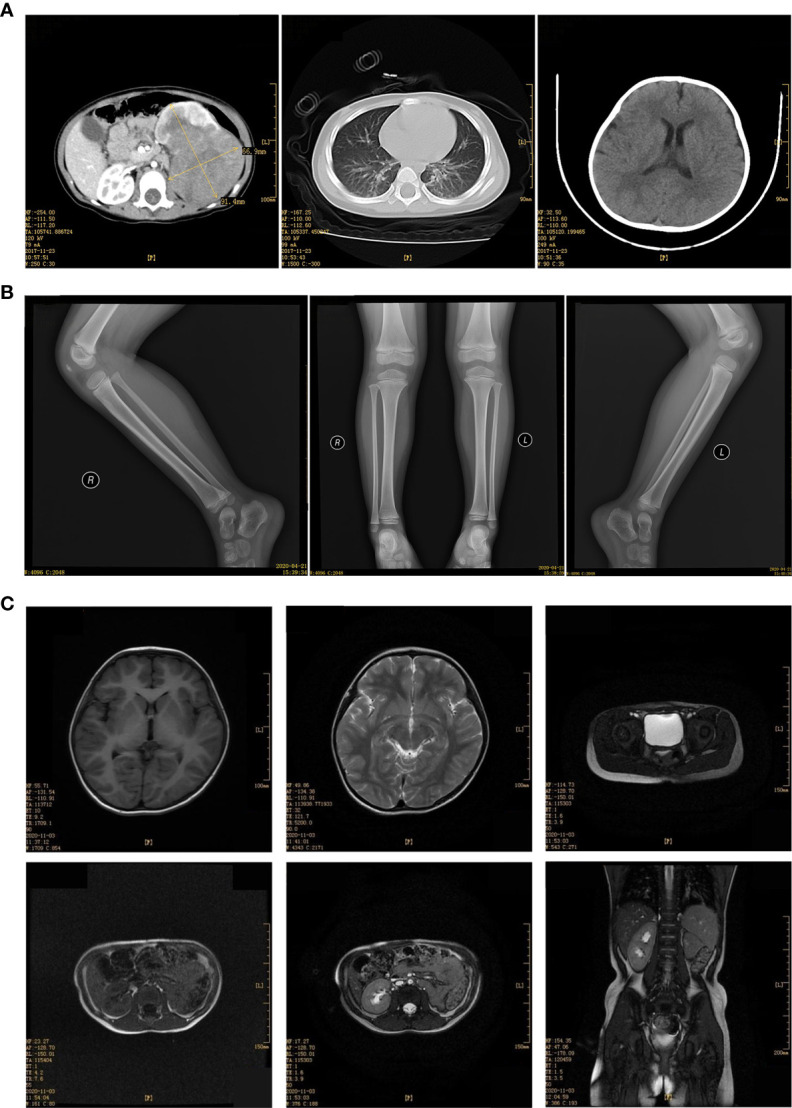
**(A)** Enhancement of CT plain scan of chest and abdomen before the operation (December 29, 2017). **(B)** Bilateral hip joint X tablets after the operation (April 21,2020). **(C)** MRI scan of the head, chest, abdomen, and pelvis after the operation (December 07, 2020).

Child 2 was a 2.1 years old girl who was admitted to the hospital on November 29, 2017, due to painless naked hematuria that lasted for more than 10 days. B-scan ultrasonography suggested left renal mass; thus, nephroblastoma was suspected. The child’s mental health, diet, sleep, and body weight were normal. No relevant physical examination and family history were reported. CT showed a large irregular mixed density (approx. 7.0 cm×8.6 cm×8.0 cm) in the left abdomen, which was further suggestive of nephroblastoma (**Figure 3A**).

The patient underwent radical resection of the left kidney on December 6, 2017. The left renal area was enlarged with solid mass (size:7.0 cm×8.6 cm×8.0 cm). The exposed part was smooth. The envelope was purplish-red and grayish-white; varicose veins were seen in the capsule; there was no obvious normal kidney tissue. Part of the macula adhered to the renal hilum and peritoneum, and varicose vein nutritional tumor tissue was visible. Along with the perirenal serosal space, the renal tumors and the upper ureter were isolated layer by layer and released the tumor body along this gap. The release included a total, complete resection of tumor tissue and ureteral stump, as well as perirenal fascia resection, abdominal para-aortic lymph node dissection. The operation was successful; intraoperative bleeding was 30 ml, and blood transfusion of 1U red blood cells was required. Operation time was 145 min.

Postoperative pathology indicated the following: the tumor cells were epithelioid, with some dense areas. The cytoplasm was vacuolated, nesting, and lamellar; dendritic blood vessels could be seen. No tumor invasion was found in the lymph nodes. Immunohistochemistry indicated FLI-1(+), CD99(+), Vimentin(+), CyclinD1(+), WT-1(+), EMA(+), Bcl2(-), Ki-67(+) 60%, CD56(-), CK(-), CD34(-), Desmin(-), and SMA(-), which suggested that renal clear cell sarcoma may not rule out renal cell carcinoma. The pathological sections were then sent to Beijing Children’s Hospital for re-examination, which indicated the following: INI-1(-), CyclinD1 (+), CK (+), EMA(+), and S-100 (+). The final diagnosis was MRTK.

The patient received the following treatment: Programme I “vincristine [VCR0.5mg/(kg·d) on day 1] + cyclophosphamide [CTX100 mg/(kg·d) on day 1-2] + actinomycin D [Act-D150μg/(kg· d) on day 1-5] + epirubicin [E-ADM 10 mg/(kg·d) on day 3-4]”. Programme II “VCR0.5mg/(kg·d) on day 1] + carboplatin [Carbopla-tin16.7 mg/(kg·d) on day 2-3] + etoposide [vp16 30 mg/(kg·d) on day 1-5]”Alternate use of the two schemes, once a day. Chemotherapy began on December 20, 2017. On December 11, 2018, the head, chest, and abdomen were examined by MRI; the hip X line did not appear abnormal on April 21, 2018 (**Figure 3B**). During the most recent follow-up on December 7, 2020, MRI suggested no metastases of the head, chest, and total abdominal and pelvic (**Figure 3C**). Currently, 3 years after surgery, the patient continues to be asymptomatic.

### 2. Effect of the Gene Expression at the Transcript Level in Patients With MRTK

#### Differential Gene (DEGs) Screening and Analysis

**Figure 4:**
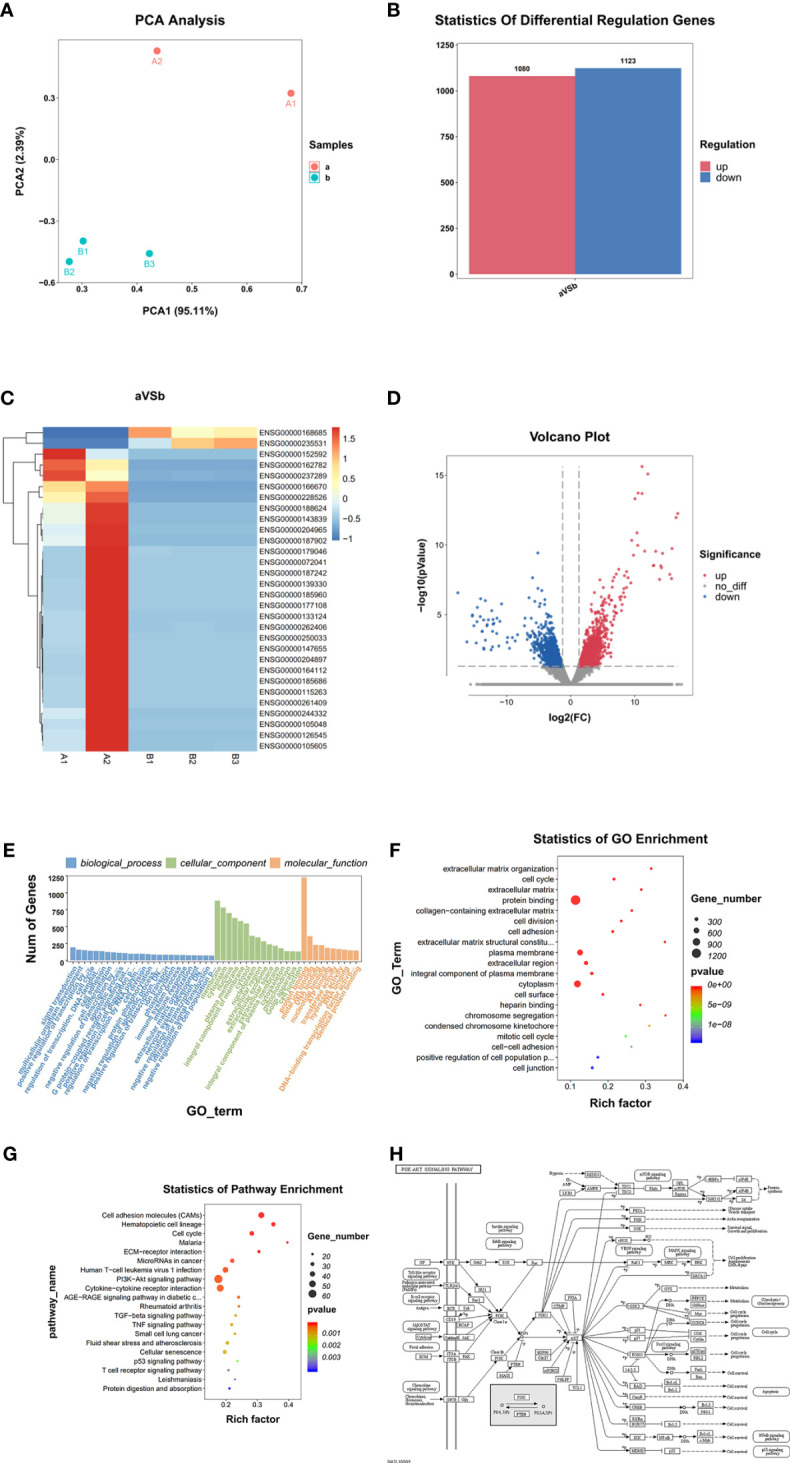
Differential gene screening and analysis. **(A)** PCA Analysis; **(B)** Differential Regulation Genes. a: Tumor tissue, B: Normal tissues; **(C)** Heat map. A1, A2: Tumor tissue (Child 1 and Child 2), B1, B2, B3: Normal tissues; **(D)** Volcano Plot. Blur: Down regulation DEGs. Red: Up regulation DEGs; **(E, F)** GO Analysis, **(G)** Statistics of Pathway Enrichment; **(H)** PI3K-AKT signaling pathway (From: http://www.kegg.jp/kegg/mapper.html).

The PCA analysis results (**Figure 4A**) showed that the overall expression of the transcriptome of MRTK pathological tissues was different compared with normal tissues. A total of 2203 DEGs were found (p-value <0.01); 1080 were up-regulated genes, and 1123 were down-regulated genes (**Figures 4B, D**). Heat map cluster analysis showed a big difference in gene expression between the normal tissues and MRTK pathological tissues. The INI1 protein corresponding gene SMARCB1 showed a significant downward trend, which is consistent with the pathology report.

#### GO Function Analysis

GO function analysis (**Figures 4E, F**) indicated that 5665 genes were involved in the biological process, 812 genes in cellular components, and 1565 genes in molecular functions. The genes involved in the protein binding function represented the highest group (1227 genes).

#### KEGG Signal Pathway Analysis

KEGG signaling pathway analysis results showed that DEGs involved in the PI3K-Akt signaling pathway (**Figure 4G**) were the most abundant, with 62 (**Figure 4H**), indicating that the PI3K-Akt signaling pathway has an important role in the occurrence and development of MRTK and microRNAs are also listed in the same position in cancer. The top 20 suggest that MicroRNA also has an irreplaceable role in the development of MRTK.

## Discussion

We believe that the above two points can be used to identify Wilms tumor during non-invasive preoperative examination. In patient 1, the tumor was 4.0 cm×3.5 cm in size. CT showed that the mass was homogeneous, which was consistent with the appearance of early small tumor foci. Also, no cyst-solid alternate dark areas characteristic of Wilms tumor were seen, which are the two main features found in postoperative pathological reports of children with renal clear cell sarcoma. The diagnosis was confirmed after consultation with the pathology department of Beijing Children’s Hospital, thus suggesting that it is difficult to distinguish the disease only from the CCSK in pathology. Experienced pathologists need to be consulted to achieve an accurate diagnosis purpose of symptomatic treatment. In addition, the most common cause of death due to MRTK is tumor metastasis. In patients with MRTK, the most common metastatic sites are the lungs and brain, followed by abdominal organs such as the liver, sacrum, and hip joints (1). The two patients’ preoperative CT showed no tumors in the lungs, brain, and liver. Moreover, X-rays of the hip and sacrococcyx showed no abnormalities. Considering that there was no metastasis, the patients were treated with radical nephrectomy.

Complete tumor resection is considered as the prerequisite and basis for long-term survival after surgery (15). It has been reported that the prognosis of children undergoing surgery after chemotherapy is worse than that of primary surgery (16). Thus, surgery should be performed as soon as possible after the discovery of MRTK. As long as the clinical metastasis is not confirmed, one or more operations should be performed as far as possible to remove all tumor tissues. If the tumor cannot be completely removed, high-dose chemotherapy and local gamma knife treatment can also improve the prognosis (17). Chemotherapy is also an important part of MRTK treatment. At present, the alternative chemotherapy regimen, including vincristine, adriamycin, cyclophosphamide, etoposide, and ifosfamide have been universally recognized by experts at home and abroad and have been suggested as a treatment for MRTK (18–20).

In this study, the two patients were 1.8 years old and 2.1 years old. No metastasis was found in the preoperative imaging examination. Both patients were treated by complete tumor resection directly. It is worth noting that patient 2 did not complete the entire chemotherapy. Radiotherapy was not added during the process, and the patient continues to be asymptomatic.

Standard chemotherapy is effective for children after MRTK. The aforementioned alternative chemotherapy regimens and doses can provide references for postoperative treatment of MRTK. Yet, it remains unclear whether there are individual differences in doses and whether chemotherapy supplemented with radiotherapy is effective for the prognosis of children with MRTK.

Compared with the normal tissue parts of children’s kidneys removed from other non-tumor diseases, RNA-seq analysis showed that children with MRTK have 2203 differential genes (DEG); 1080 DEGs were up-regulated, and 1123 DEGs were down-regulated. Moreover, the GO analysis results showed that most DEGs were involved in protein binding (up to 1227 genes) (**Figures 4B, D**). This result suggests that many proteins involved in protein binding are included in the development of MRTK. Many DEGs were also involved in membrane composition, thus suggesting that cell membrane constituent proteins may be used as new therapeutic targets when treating patients with MRTK. These data suggested that the MRTK pathogenic genes and cancer-causing genes were down-regulated, which further proved the correctness of the previous diagnosis of the patient’s condition, and also suggested that our subsequent research and analysis are true and reliable (**Table 1**).

**Table 1:** MRTK pathogenic genes and cancer-causing genes.

gene_name	gene_id	transcript_id	Description	trans_type	FPKM.A1	FPKM.A2	FPKM.B1	FPKM.B2	FPKM.B3	fc	regulation	chr	start	end	strand
MRTK pathogenic genes															
SMARCB1	ENSG00000099956	ENST00000407422;ENST00000644036;ENST00000344921;ENST00000646421;ENST00000491967;ENST00000647057;ENST00000644619;ENST00000263121;ENST00000407082;ENST00000417137;ENST00000646723;ENST00000643421;ENST00000646911;ENST00000644462;ENST00000634926;ENST00000635578;ENST00000642727;ENST00000644467;ENST00000642275;ENST00000477836;ENST00000645799	SWI/SNF related, matrix associated, actin dependent regulator of chromatin, subfamily b, member 1 [Source:HGNC Symbol;Acc:HGNC:11103]	protein_coding;retained_intron;nonsense_mediated_decay	1.22580511878971	0.766276115400908	2.17320733276853	2.28854727842663	1.89086102263238	0.47037661705428	down	chr22	23786931	23838008	+
Cancer-causing genes(Cancer core genes (list only the top five)															
TACSTD2	ENSG00000184292	ENST00000371225	tumor associated calcium signal transducer 2 [Source:HGNC Symbol;Acc:HGNC:11530]	protein_coding	266.599854	0.189811	1.935009	10.726301	0.628289	30.1126089282303	up	chr1	58575423	58577773	–
PTTG1	ENSG00000164611	ENST00000524244;ENST00000352433;ENST00000517480;ENST00000523659;ENST00000520452;ENST00000393964;ENST00000519287	pituitary tumor-transforming 1 [Source:HGNC Symbol;Acc:HGNC:9690]	retained_intron;protein_coding;processed_transcript	3.87797038936709	21.4844095782278	1.58469719594937	1.86563797670886	0.870966115443038	8.80373003755733	up	chr5	160421855	160428739	+
TPD52	ENSG00000076554	ENST00000379096;ENST00000448733;ENST00000518937;ENST00000517427;ENST00000520527;ENST00000379097;ENST00000517462;ENST00000519303;ENST00000523395;ENST00000523193;ENST00000523319;ENST00000521354;ENST00000520877;ENST00000521618;ENST00000518517;ENST00000524194;ENST00000521241;ENST00000517445;ENST00000523753;ENST00000522364;ENST00000519250;ENST00000518500;ENST00000520795;ENST00000520741;ENST00000521561;ENST00000523783;ENST00000523564;ENST00000520035;ENST00000602950	tumor protein D52 [Source:HGNC Symbol;Acc:HGNC:12005]	protein_coding;nonsense_mediated_decay;processed_transcript;retained_intron	3.78222204660242	3.62655071721361	0.810742042210565	0.865708026595096	0.647882930828352	4.78122504282902	up	chr8	80034745	80231172	–
TUSC3	ENSG00000104723	ENST00000503191;ENST00000382020;ENST00000515859;ENST00000506802;ENST00000510836;ENST00000509380;ENST00000503731;ENST00000507400;ENST00000509177;ENST00000511783;ENST00000508446;ENST00000507316;ENST00000511342	tumor suppressor candidate 3 [Source:HGNC Symbol;Acc:HGNC:30242]	processed_transcript;protein_coding;nonsense_mediated_decay;retained_intron	15.8984117990366	18.1366191400771	6.34476836088632	6.80686292697495	5.8215049495183	2.69078057364551	up	chr8	15417215	15766649	+
TP53	ENSG00000141510	ENST00000413465;ENST00000635293;ENST00000359597;ENST00000504290;ENST00000504937;ENST00000510385;ENST00000610623;ENST00000618944;ENST00000619186;ENST00000610292;ENST00000269305;ENST00000620739;ENST00000617185;ENST00000420246;ENST00000455263;ENST00000610538;ENST00000622645;ENST00000445888;ENST00000619485;ENST00000576024;ENST00000615910;ENST00000509690;ENST00000514944;ENST00000574684;ENST00000505014;ENST00000508793;ENST00000604348;ENST00000503591;ENST00000571370	tumor protein p53 [Source:HGNC Symbol;Acc:HGNC:11998]	protein_coding;nonsense_mediated_decay;processed_transcript;retained_intron	9.99790931997187	11.5173892960619	3.87862323136428	4.47784009388186	4.37257840963432	2.53537922148652	up	chr17	7661779	7687538	–

Through the analysis of the KEGG signaling pathway, we found that up to 62 genes were involved in the PI3K-AKT signaling pathway (**Figure 4G**), which suggested that the PI3K-AKT signaling pathway has a vital role in the occurrence and development of MRTK (**Figure 4H**). More importantly, this is the first time that the signal pathway has been involved in MRTK. Various growth factors/ligands specific to fibroblast growth factors of different receptor tyrosine kinases (RTKs) can activate PI3-kinase (21). Growth factors mediate PI3K activation by stimulating RTK. Akt is the main mediator of the PI3K pathway. It interacts with PDK1 (phosphoinositide-dependent kinase 1) to cause Akt phosphorylation at breast cancer to play a role (22). A number of studies have shown that the PI3K/Akt pathway in human cancers, especially the expression of the two important genes PIK3CA and PTEN, usually significantly changes in human cancers, which have been found in more than 70% of tumor types (23, 24).

PI3K-Akt has a certain role in cell proliferation, apoptosis, migration, and other physiological activities. Its activity level is one of the key factors affecting the physiological activities of cancer cells. At present, more than 20 PI3K and Akt inhibitors have entered the stage of clinical trials (25). Further in-depth studies of the PI3K-Akt signaling pathway mechanism are necessary to gain a more comprehensive understanding of the formation and evolution of tumors to treat cancer diseases.

Among the other top 20 high-abundance signal pathways shown by KEGG, most of them are very common tumor signal pathways, such as cell cycle, calcium signal pathway, etc. (26–28). However, so far, no study has examined the changes in microRNA in MRTK cells. The KEGG results in this study suggest that many microRNA-related genes (up to 33) have changed in MRTK. Although most of them have been reported in other diseases, these microRNAs have never been deeply investigated in MRTK. Interestingly, these genes have been proven to be extremely safe and effective new targets with considerable potential in other tumors (29–31).

Although this study provides many new diagnostic and therapeutic targets with greater feasibility, further in-depth experimental research is still needed. We plan to further explore the new targets’ feasibility and safety in subsequent clinical treatment and basic research.

Because children’s MRTK accumulates throughout the whole kidney, it is difficult to obtain satisfactory paracancerous tissues. Therefore, it is possible to select the normal tissue part of the pathological kidney tissue in non-tumor patients with no other symptoms to replace the adjacent or normal kidney tissue only through pathology identification.

## Conclusion

Our data suggest that the PI3K-Akt signaling pathway and microRNA-related proteins as targets have extremely high potential value for the diagnosis and treatment of MRTK.

## Limitations and Strengths

This study has a few limitations. The sample size is too small to identify key genes in this signaling pathway associated with MRTK disease.

The research was done through high-throughput RNA sequence analysis, and the results and information were complete with high-quality. With the improvement of high-throughput sequencing, its higher recognition accuracy and sensitivity greatly reduced the error caused by too few experimental samples. Therefore, the evaluation of signal pathways in this study is representative.

## Data Availability Statement

The datasets presented in this study can be found in online repositories. The names of the repository/repositories and accession number(s) can be found below: GEO Submission (GSE167547, link: https://www.ncbi.nlm.nih.gov/geo/query/acc.cgi?acc=GSE167547).

## Ethics Statement

The study was approved by the Ethics Committee of Kunming Children’s Hospital. Written informed consent to participate in this study was provided by the participants’ legal guardian/next of kin. Written informed consent was obtained from the minor(s)’ legal guardian/next of kin for the publication of any potentially identifiable images or data included in this article.

## Author Contributions

CZ and SC: study design, data collection and analysis, statistical analysis, and manuscript drafting. LL: study design, and data collection and analysis. JL: study design and data collection. HT, XH, and YX: data collection. ZY: manuscript revision. BY: study design and critical revision of the manuscript. LD: study design and manuscript revision. All authors contributed to the article and approved the submitted version.

## Funding

This study was supported by the Yunnan Education Department of Science Research Fund (No.:2020 J0228), Kunming City Health Science and Technology Talent “1000” training Project (No.:2020- SW (Reserve)-112), Kunming Health and Health Commission Health Research Project (No.:2020-0201-001), Kunming Xishan District Science and Technology Project (No.:2020-Xike word 23), Kunming Medical Joint Project of Yunnan Science and Technology Department (No.:202001 AY070001-271).

## Conflict of Interest

The authors declare that the research was conducted in the absence of any commercial or financial relationships that could be construed as a potential conflict of interest.
